# An Assessment of Public Awareness Regarding Pancreatitis: A Cross-Sectional Study in the Eastern Province of Saudi Arabia

**DOI:** 10.7759/cureus.71483

**Published:** 2024-10-14

**Authors:** Latifah K Alnami, Fatimah Alghannam, Atheer A Alalaiwi, Abdul Qadeer Memon, Zeyad K Al shehri, Layan Asiri, Abdulrahman A Alhawas, Abdullah A Alahmad, Watan A Alsahlawi, Ali E Alawsi

**Affiliations:** 1 Internal Medicine, King Faisal University, Al-Ahsa, SAU; 2 Internal Medicine, Hafr Albatin University, Hafr Albatin, SAU; 3 General Surgery, King Faisal University, Al-Ahsa, SAU

**Keywords:** acute pancreatitis, awareness, chronic pancreatitis, knoweldge, pancreatitis

## Abstract

Introduction: Pancreatitis, characterized by abdominal pain and elevated pancreatic enzymes, remains a significant public health issue. This study aims to assess the awareness of the general population in the Eastern Province of Saudi Arabia regarding pancreatitis, focusing on symptoms, risk factors, and management.

Methodology: A cross-sectional study was conducted using a structured questionnaire distributed to 446 participants through social media. Data were analyzed using descriptive statistics and Chi-square tests.

Results: The study revealed that the most recognized symptoms of pancreatitis were abdominal pain, nausea, and vomiting. High-fat diets and genetic factors were the most commonly acknowledged risk factors. Medications and dietary changes were the most frequently known treatment methods. The overall knowledge level was 46%, with a significant difference in knowledge between healthcare and non-healthcare personnel. However, there were no significant differences in knowledge based on gender or income levels.

Conclusion: The study highlights a considerable gap in public awareness of pancreatitis, emphasizing the need for targeted educational initiatives. Enhanced public health campaigns could improve early detection and management, potentially reducing the disease burden in Saudi Arabia.

## Introduction

Pancreatitis, an inflammation of the pancreas, can be acute or chronic, characterized by abdominal pain and elevated pancreatic enzymes. Acute pancreatitis (AP) is a sudden inflammation, often severe, while chronic pancreatitis (CP) is a long-standing inflammation altering pancreatic structure and function [1]. Globally, the incidence of AP is estimated at 34 cases per 100,000 population annually, while available evidence suggests that the incidence of CP is around 25 cases per 100,000 individuals, with a prevalence of approximately 92 cases per 100,000 individuals [2,3]. In Saudi Arabia, the incidence is 7.2 cases per 100,000 population. This incidence rate was based on the number of new cases identified at the single center during the study period [4].

CP and AP share several risk factors, which include gallstones, chronic alcohol consumption, hypertriglyceridemia, and the use of certain medications. Additionally, recurrent episodes of acute pancreatitis are recognized as a significant risk factor for the development of chronic pancreatitis, as repeated inflammation can lead to permanent pancreatic damage and fibrosis [5-8]. Management strategies range from supportive care in mild cases, such as fluid resuscitation, pain management, and nutritional support, to more targeted interventions, such as addressing the underlying causes and managing complications. In severe cases of both AP and CP, surgical or endoscopic interventions may be necessary, including drainage of necrosis or abscesses in AP or surgical decompression or resection in CP to manage pain or complications [9-12]. The literature emphasizes the need for public awareness about these risk factors and early symptoms for better prognosis and management [13]. A study conducted in Tabuk, Saudi Arabia, showed that participants lacked sufficient knowledge about the risk factors and symptoms of pancreatitis [14]. Another study in Saudi Arabia, conducted among university students, found that students were unfamiliar with the fundamental interventional knowledge of Acute Pancreatitis [15]. Despite significant advancements in understanding pancreatitis, awareness in regions like Saudi Arabia remains limited. This study aims to assess the awareness of the general population in the Eastern Province of Saudi Arabia regarding pancreatic inflammatory disorders, including both acute and chronic pancreatitis, with a focus on risk factors, symptoms, and management.

## Materials and methods

Study design

This cross-sectional study was conducted in the Eastern Province of Saudi Arabia. It involved a structured questionnaire distributed to the general population to assess their awareness of the risk factors and management of pancreatitis.

Study population

Participants were selected using a stratified random sampling method to ensure representation from different demographic groups, including age, gender, and educational background. The target sample size was 446 respondents.

Inclusion and exclusion criteria 

The inclusion criteria for this study were as follows: participants had to be residents of the Eastern Province of Saudi Arabia, aged 18 years and older, and willing to provide informed consent. Both healthcare and non-healthcare workers were included to ensure a diverse representation of the general population. Participants needed to have access to the online survey platform to complete the questionnaire.

The exclusion criteria were individuals under the age of 18 years, non-residents of the Eastern Province, and those who were unable or unwilling to provide informed consent. Additionally, participants who had incomplete or duplicate survey responses were excluded from the analysis to ensure the integrity of the data.

Data collection

Data were collected through an online survey platform. The questionnaire was distributed via social media, email, and community centers to ensure a wide reach. The survey remained open for responses for one month.

Questionnaire

The questionnaire was divided into three sections: the first section covered demographic information, including age, gender, education level, and occupation; the second section assessed knowledge of pancreatitis with questions about awareness of the pancreas, symptoms of pancreatitis, and understanding of its risk factors; and the third section focused on the management of pancreatitis, addressing knowledge of treatment options, preventive measures, and sources of information about the disease.

Data analysis

Descriptive statistics were used to summarize the demographic data and responses. Chi-square tests were employed to analyze the association between demographic variables and awareness levels. Statistical significance was set at p < 0.05.

Ethical considerations

The study was conducted in accordance with ethical guidelines. Informed consent was obtained from all participants, and the confidentiality of the data was maintained. The study protocol was reviewed and approved by the Institutional Review Board (IRB) of King Faisal University with ethical approval code KFU-REC-2024-SEP- ETHICS254.

## Results

The study included 446 participants, ranging in age from 18 to 70 years, with a mean age of 39 (Tables 1, 2). The majority of the respondents were females 280 (62.8%), while 166 (37.2%) were males. Most participants, 381 (85.4%) were non-healthcare employees, with only 65 (14.6%) employed in healthcare-related fields. In terms of income distribution, the majority 274 (61.4%) reported a monthly income between 5,000 and 10,000 SR, while 84 (18.8%) earned between 10,001 and 15,000 SR, 52 (11.7%) between 15,001 and 20,000 SR, and 36 (8.1%) reported earning more than 20,000 SR. 

**Table 1:** Demographic chrematistics

Variables	Categories		N = 446	%
Gender	Male		166	37.2
	Female		280	62.8
Occupation status	Employed healthcare provider		65	14.6
	Employed non-healthcare provider		381	85.4
Monthly income	From 5000 to 10000 SR		274	61.4
	From 10001 to 15000 SR		84	18.8
	From 15001 to 20000 SR		52	11.7
	More than 20000 SR		36	8.1

**Table 2:** Age distribution

Variables	Min	Max	Mean	SD
Age	18	70	39	±13.8

The results indicate that the most common source of information about pancreatitis was university education 57 (38%), followed by internet or online sources 47 (31.3%) (Table 3). Family and friends contributed 25 (16.7%) of the information sources, while healthcare providers accounted for 21 (14%).

**Table 3:** Sources of information about pancreatitis

Source	N	%
University	57	38
Family/friends	25	16.7
Internet/online sources	47	31.3
Healthcare provider	21	14
Total	150	100

The results indicated that the most effective methods to increase awareness about pancreatitis and its risk factors, from the participants' perspective, were word of mouth 315 (70.6%), followed by TV/radio ads 287 (64.3%), short movies/animations 217 (48.7%), and informational brochures/pamphlets 128 (28.7%) (Table 4).

**Table 4:** The methods could be used to increase awareness about pancreatitis and its risk factors

No	Methods	N	%
1	Informational brochures/pamphlets	128	28.7
2	Short movies/animations	217	48.7
3	Word of mouth	315	70.6
4	TV/radio ads	287	64.3

The results indicated that the most commonly known symptom of pancreatitis was abdominal pain 227 (50.9%), followed by nausea and vomiting 211 (47.3%), diabetes 210 (47.1%), indigestion 135 (30.3%), fever 110 (24.7%), and rapid pulse 63 (14.1%) (Table 5).

**Table 5:** The symptoms of pancreatitis

No	Symptoms	N	%
1	Abdominal pain	227	50.9
2	Nausea and vomiting	211	47.3
3	Fever	110	24.7
4	Rapid pulse	63	14.1
5	Indigestion	135	30.3
6	Having diabetes	210	47.1
7	I don't know	85	19.1

The results indicated that the most commonly known risk factor for pancreatitis was a high-fat diet 279 (62.6%), followed by genetic factors 217 (48.7%), alcohol consumption 175 (39.2%), medications 154 (34.5%), gallstones 143 (32.1%), and infections 64 (14.3%) (Table 6).

**Table 6:** The risk factors for pancreatitis

No	Risk factors	N	%
1	Alcohol consumption	175	39.2
2	Gallstones	143	32.1
3	High-fat diet	279	62.6
4	Genetic factors	217	48.7
5	Medications	154	34.5
6	Infections	64	14.3
7	I don't know	111	24.9

The results indicated that the most commonly recognized treatment for pancreatitis was medications 294 (65.9%), followed by dietary changes 225 (50.4%) and surgery 119 (26.7%). Additionally, 16 (3.6%) of participants believed that no treatment was required (Table 7).

**Table 7:** The treatment of pancreatitis

No	Methods	N	%
1	Surgery	119	26.7
2	Dietary changes	225	50.4
3	Medications	294	65.9
4	No treatment required	16	3.6
5	I don't know	110	24.7

The results demonstrated that the overall knowledge level regarding pancreatitis was 46%. The item with the highest level of knowledge was “Increased awareness of pancreatitis and its risk factors would lead to early detection and prevention” (85%), followed by “Regular check-ups can help in the early detection and management of pancreatitis” (79%). Conversely, the items with the lowest levels of knowledge were “Have you or someone you know ever been diagnosed with pancreatitis” (17%) and “Are there any traditional or home remedies that you believe can help manage pancreatitis” (14%) (Table 8).

**Table 8:** The knowledge level of pancreatitis

No	Items	Mean ±SD	%
1	Aware of the pancreas and its functions	0.73±0.44	73
2	Receiving any information about pancreatitis and its risk factors	0.34±0.47	34
3	Increased awareness of pancreatitis and its risk factors would lead to early detection and prevention	0.85±0.36	85
4	knowing any preventive measures for pancreatitis	0.19±0.40	19
5	The rate of knowledge of pancreatitis	2.15±1.11	43
6	The most age groups are at risk for developing pancreatitis	0.36±0.48	36
7	Have you or someone you know ever been diagnosed with pancreatitis	0.17±0.38	17
8	There are any traditional or home remedies that you believe can help manage pancreatitis	0.14±0.35	14
9	The regular check-ups can help in the early detection and management of pancreatitis	0.79±0.41	79
10	What role do you think diet plays in the prevention and management of pancreatitis	0.73±0.44	73
	Total	6.45±2.42	46

The results indicated a significant difference in knowledge levels between employed healthcare providers and non-healthcare providers, with a t-value of 4.74 and a p-value less than 0.001. Employed healthcare providers had a higher mean knowledge level (7.74) compared to non-healthcare providers (6.23). However, there was no significant difference in knowledge levels between males and females (p = 0.316). Additionally, knowledge levels did not significantly vary with monthly income (F = 1.65, p = 0.178) (Table 9).

**Table 9:** The difference in the knowledge level due to demographic chrematistics

Variables	Categories	Mean	Test	Statistics	P-value
Gender	Male	6.61	Independent Samples Test	1.005	0.316
	Female	6.36			
Occupation status	Employed healthcare provider	7.74	Independent Samples Test	4.74	< 0.001
	Employed non-healthcare provider	6.23			
Monthly income	From 5000 to 10000 SR	6.51	ANOVA	1.65	0.178
	From 10001 to 15000 SR	6.40			
	From 15001 to 20000 SR	5.87			
	More than 20000 SR	6.97			

## Discussion

The findings of this study highlight a significant gap in awareness and knowledge of pancreatitis among the general population in the Eastern Province of Saudi Arabia. The results demonstrated that the primary source of information about pancreatitis was university education, followed by internet/online sources. The most recognized symptom of pancreatitis was abdominal pain, with nausea and vomiting. The predominant risk factor was a high-fat diet, with genetic factors. Medications were the most well-known treatment for pancreatitis, followed by dietary changes. A significant difference in knowledge levels was observed between employed healthcare providers and non-healthcare providers. No significant differences were found in knowledge levels based on gender or monthly income.

Comparing the findings of this study with existing literature reveals both similarities and differences in awareness and knowledge of pancreatitis across various populations. Two studies conducted by Jastaniah et al. (2012) [15] and Patil et al. (2018) [16], for instance, also identified a general lack of public knowledge about pancreatitis, with abdominal pain and nausea being the most recognized symptoms, much like in our study. This similarity could be due to the common presentation of these symptoms in pancreatitis across different populations, making them more easily recognizable even among those with limited knowledge of the disease. Another study by Alalawi et al. (2023) found that the most common sources of information about pancreatitis were educational seminars, which aligns with the role of university education as the primary source identified in our research [14]. This difference might be attributed to regional variations in how health information is disseminated, where in some areas, universities may play a more significant role in public health education, while in others, healthcare providers and digital platforms may be more influential [17].

The recognition of high-fat diets as a risk factor for pancreatitis contrasts with findings from Jastaniah et al. (2012), who highlighted the importance of alcohol in the etiology of pancreatitis [15]. This difference likely reflects variations in the emphasis placed on different lifestyle factors across studies, although both dietary habits and alcohol consumption are universally recognized as contributing to the development of pancreatitis in different health systems. However, the emphasis on genetic predisposition as a significant risk factor in our study appears to be more pronounced compared to findings in other studies, which have suggested that lifestyle factors are more commonly recognized than genetic factors in the general population [7-8,18]. This difference could be due to varying public awareness or the prevalence of genetic conditions in different regions, suggesting that in some populations, genetic risk factors might be more emphasized or more relevant.

Moreover, the significant difference in knowledge levels between healthcare providers and non-healthcare providers observed in our study aligns with the research by Su et al. (2024), which also demonstrated that medical professionals have a better understanding of pancreatitis compared to the general public [19]. This similarity is expected, as healthcare providers are typically more exposed to medical education and clinical experiences that enhance their knowledge of specific conditions like pancreatitis. On the other hand, our study found a significant difference in knowledge levels between healthcare providers and non-healthcare providers, similar to García-García and Pérez-Rivas (2022), who also identified employment as a key factor influencing health literacy [20]. However, both studies showed no significant difference in knowledge based on gender or income, indicating these factors have less impact on health literacy.

Limitation and recommendation 

This study has several limitations, including its reliance on a specific demographic and geographic region, which may limit the generalizability of the findings to the broader population of Saudi Arabia. Additionally, the use of a cross-sectional design prevents the assessment of causality between awareness levels and actual health outcomes related to pancreatitis. The questionnaire-based approach also introduces the possibility of response bias, as participants may overestimate or underestimate their knowledge. Furthermore, the study's reliance on self-reported data may affect the accuracy of the results. To address these limitations, future research should consider a larger and more diverse sample size across different regions of Saudi Arabia, utilize longitudinal designs to better understand the impact of awareness on health behaviors, and incorporate more objective measures of knowledge and awareness. Additionally, targeted educational programs should be developed to enhance public awareness, especially focusing on populations with lower levels of knowledge, such as non-healthcare workers, to improve early detection and management of pancreatitis.

## Conclusions

This study reveals a significant gap in the general population's awareness and knowledge of pancreatitis in the Eastern Province of Saudi Arabia. Despite the prevalence of the disease, the overall knowledge level remains low, particularly regarding symptoms, risk factors, and treatment options. The findings underscore the necessity for targeted educational interventions to improve public understanding of pancreatitis, potentially leading to better disease outcomes. The study also highlights the importance of healthcare providers in disseminating accurate information and the need for consistent messaging across all demographic groups.
